# Trusted to share or tempted to hoard? Unpacking employee knowledge hiding through the interplay of leader trust and knowledge psychological ownership

**DOI:** 10.3389/fpsyg.2025.1659249

**Published:** 2025-10-10

**Authors:** Wei Zhang, Shunying Leng, Hao Ran

**Affiliations:** School of Economics and Management, Yunnan Minzu University, Kunming, China

**Keywords:** leader trust, job satisfaction, knowledge hiding, knowledge psychological ownership, conservation of resources theory, social exchange theory

## Abstract

**Introduction:**

Anchored in Social Exchange Theory, Conservation of Resources Theory, Affective Events Theory, and Psychological Ownership Theory, this study develops an integrated model linking leader trust, job satisfaction, and knowledge hiding, while positioning knowledge psychological ownership (KPO) as a contextual boundary condition.

**Methods:**

Survey data were collected from 518 matched leader-employee dyads across 17 Chinese knowledge-intensive firms in IT services, pharmaceutical R&D, high-end equipment manufacturing, and financial consulting. Structural equation modeling combined with PROCESS bootstrap analysis was employed to test the hypothesized relationships.

**Results:**

Confirmatory-factor-analysis results indicate satisfactory discriminant validity for all four focal constructs. Empirical evidence shows that: (1) leader trust significantly curbs employee knowledge hiding (β = −0.31, *p* < 0.001); (2) job satisfaction partially mediates this relationship, with the indirect path accounting for 34 per cent of the total effect (β = 0.46, *p* < 0.001); and (3) KPO exerts a significant negative moderating influence on both the “leader trust → job satisfaction” path and the overall indirect effect, reducing the mediation coefficient from −0.17 to −0.06 under high-KPO conditions (β = −0.23, *p* < 0.001). These findings remain robust after controlling for organizational support, team competition, and industry heterogeneity.

**Discussion:**

The study enriches the antecedent framework of knowledge hiding by foregrounding vertical trust, illuminating the dynamic tension between reciprocity motivation and resource-defence motivation, and clarifying the double-edged boundary role of psychological ownership. Practically, Organizations should enhance perceived trust through empowerment and feedback while implementing monitoring systems to cultivate knowledge-sharing climates.

## 1 Introduction

As the global economy shifts from a resource-driven to a knowledge-driven paradigm, competitive advantage depends less on tangible inputs such as capital and land and more on knowledge assets that are difficult to imitate or transfer (Argote and Ingram, 2000). The widespread deployment of big data, artificial intelligence, and cloud computing has accelerated both the speed and density of knowledge flows, making knowledge management a core function for sustaining long-term competitiveness. However, the rapid circulation of knowledge also brings a paradox: while firms can benefit from knowledge sharing, they simultaneously face the challenge that individuals may deliberately conceal or withhold knowledge. In modern firms-where individual expertise differentiation and performance contests are salient-employees often hesitate, or even refuse, to share their unique know-how. When confronted with knowledge requests from colleagues or teams, employees may deliberately withhold information by feigning ignorance, offering excuses, or invoking confidentiality. This intentional refusal is conceptualized as knowledge hiding (Connelly et al., 2012). Extensive empirical research indicates that knowledge hiding not only undermines team innovation and prolongs new-product development cycles but also erodes organizational trust and employee satisfaction (Cerne et al., 2012; Zhou et al., 2016).

Early studies treated knowledge hiding as the antithesis of knowledge sharing and concentrated on horizontal antecedents-such as interpersonal distrust, team competition climate, or individual personality traits (Peng, 2013; Malik et al., 2019). However, in China's high-power-distance, relationship-oriented context, employees are acutely sensitive to their direct supervisor's attitudes and evaluations. The extent to which leaders trust subordinates may therefore constitute a critical cue in weighing the risks and benefits of sharing. Although a number of studies have examined interpersonal trust, systematic evidence on how vertical trust-particularly leader trust-affects knowledge hiding remains limited and fragmented, with most prior work focusing only on peer-level dynamics or treating leader trust as a background factor (Dirks and Ferrin, 2002). Drawing on Social Exchange Theory (SET), employees who perceive strong endorsement of their competence and integrity from leaders are motivated to reciprocate positively. Conservation of Resources Theory (COR) further posits that vertical trust provides socio-emotional and status resources that buffer the resource-loss anxiety associated with knowledge leakage (Hobfoll et al., 2018).

Affective Events Theory (AET) adds that positive workplace events first influence employees' emotional states and subsequently shape their work attitudes and behaviors. Leader trust, as a salient affective event, is expected to elevate job satisfaction, which is closely linked to pro-organizational behavior. Whether job satisfaction mediates the leader-trust → knowledge-hiding link, however, remains under-examined. Simultaneously, the Psychological Ownership Framework (POF) cautions that once employees develop a sense of “this is mine” toward a resource, they tend to guard it to protect personal boundaries (Pierce et al., 2003). When employees hold strong Knowledge Psychological Ownership (KPO) over their expertise, they may fear that sharing will diminish their advantage, thereby attenuating the positive influence of leader trust. KPO is thus likely to be a crucial boundary condition in the trust-knowledge-hiding pathway.

**Figure 1:**
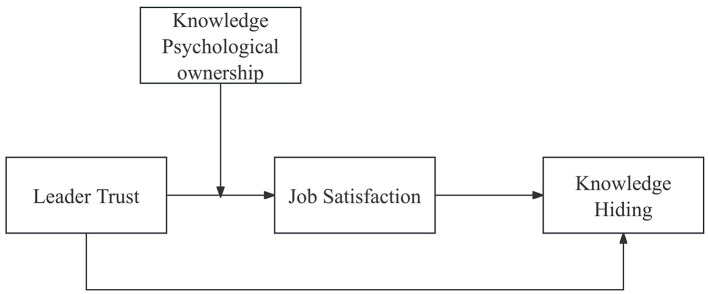
Theoretical framework.

Against this theoretical and practical backdrop, the present study addresses three research questions: (1) Can leader trust significantly suppress knowledge-hiding behavior? (2) Does job satisfaction serve as an intermediary between leader trust and knowledge hiding? (3) In what manner does KPO intervene in this causal chain? Figure 1 illustrates the conceptual framework underlying these questions. By tackling these questions, we aim to enrich the literature on vertical trust and knowledge hiding by illuminating the interaction between affective channels and resource-conservation motives, and to offer actionable guidance for knowledge-intensive enterprises seeking a balance between the virtuous cycle of “trust-satisfaction-sharing” and the vicious cycle of “possession-defense-hiding.”

## 2 Literature Review

### 2.1 Leader Trust: Conceptualization and Mechanisms

Within an organizational context, leader trust refers to the extent to which subordinates perceive their immediate manager to be reliable in terms of ability, integrity and benevolence (Whitener et al., 1998; Gillespie, 2003). A robust body of evidence demonstrates that leader trust diminishes vertical communication barriers and enhances organizational citizenship behavior and team performance (Dirks and Ferrin, 2002; Wei and Long, 2009; Schaubroeck et al., 2011; Knoll and Gill, 2011). From the perspective of Social Exchange Theory (Blau, 1956), a high level of trust constitutes a social resource conferred by leaders, thereby motivating subordinates to reciprocate through loyalty, voice and innovative conduct.

The influence of leader trust on knowledge-related behaviors, however, remains equivocal. In the hospitality sector, Fu and Zhong (2020) found that leader trust buffers the negative impact of psychological contract breach on knowledge sharing, whereas a study of high-technology firms by Lu et al. (2021) showed that perceived distrust from leaders significantly curtails employees' innovation effort and knowledge sharing. Taken together, these findings suggest that leader trust may not exert a uniform effect; rather, its impact depends on contextual characteristics and employees' cognitive appraisals. Conservation of Resources Theory (Hobfoll, 1989) further posits that trusted employees view such trust as a valuable socio-emotional resource and are therefore willing to deploy additional “available resources” in exchange; conversely, when trust is absent, they are inclined to conserve residual resources by withholding knowledge to avoid potential losses. Consequently, it is essential to examine both the direct and indirect effects of leader trust on knowledge hiding across diverse contexts and to identify the boundary conditions that shape these relationships.

### 2.2 Knowledge Hiding: Measurement, Antecedents, and Research Gaps

Since Connelly et al. (2012) introduced a 12-item instrument encompassing three forms of knowledge hiding-playing dumb, evasive hiding, and rationalized hiding-the literature on this construct has expanded rapidly. Empirical studies have identified antecedents at the individual, interpersonal, and organizational levels. At the individual level, traits such as Machiavellianism, low self-efficacy, and knowledge uniqueness are positively associated with hiding behavior (Argote and Ingram, 2000; Lin and Huang, 2010; Anand and Jain, 2014). At the interpersonal level, interpersonal distrust and a competitive team climate significantly increase the propensity to withhold knowledge (Malik et al., 2019; Jiang and He, 2013). At the organizational level, territorial culture, performance pressure, and uncertainty surrounding intellectual-property rights have all been shown to trigger knowledge hiding (He and Jiang, 2016).

Despite these insights, prior studies seldom address intra-team heterogeneity-that is, why some employees engage in hiding while others share knowledge under the same conditions. In particular, the interplay between leader trust and employees' subjective appraisals, such as knowledge psychological ownership, has been under-examined. Moreover, most existing studies are cross-sectional, providing little insight into the temporal dynamics of knowledge hiding or its long-term performance consequences. Longitudinal designs and mixed-methods approaches are therefore needed to illuminate the developmental trajectory of knowledge hiding and to capture its evolving impact on organizational outcomes.

### 2.3 Job Satisfaction: A Potential Bridge between Trust and Knowledge Behavior

Job satisfaction is commonly defined as employees' global affective evaluation of their work tasks, environment, remuneration, and developmental opportunities (Hoppock, 1935). From a Conservation of Resources perspective, job satisfaction reflects a favorable appraisal of the resources that the organization provides, thereby attenuating employees' need to defensively guard their own resources-particularly knowledge. Prior research demonstrates that job satisfaction is shaped by organizational identification and fosters belongingness, stimulates positive discretionary behavior, and ultimately improves employees' evaluation of the organization (Bartels et al., 2006; Uddin et al., 2021). Among knowledge workers, higher satisfaction strengthens the motivation to reciprocate organizational support (Chen and Wang, 2019). However, evidence remains scarce regarding whether job satisfaction can dampen knowledge hiding and how it mediates the link between leader trust and knowledge-related behavior. Clarifying this pathway will enrich theoretical understanding and provide actionable insights for organizations seeking to reduce knowledge loss through enhancing employee satisfaction.

### 2.4 Knowledge Psychological Ownership: Boundary Regulation and a Double-Edged Sword

Psychological ownership refers to a subjective sense of “mine” that is psychological rather than legal in nature (Pierce et al., 2003). Extending this notion, Knowledge Psychological Ownership (KPO) captures employees' perceived control and proprietary claims over the knowledge they possess (Peng, 2013). Empirical studies indicate that employees with high KPO are more inclined to engage in territorial behaviors and knowledge-hiding strategies (Liu et al., 2013). Conversely, some scholars argue that in contexts of strong leader trust or high job satisfaction, KPO can be re-channeled into a positive impetus for knowledge sharing. Accordingly, KPO may function as a boundary condition in the leader-trust-knowledge-hiding linkage: when employees feel deeply trusted by their leader and are highly satisfied, KPO's defensive effect is attenuated; when trust and satisfaction are weak, KPO can intensify the urge to hoard knowledge. This double-edged nature of KPO has yet to be thoroughly validated, calling for multi-context, multi-level investigations to clarify its contingent role.

## 3 Research hypotheses

Integrating Conservation of Resources theory (COR; Hobfoll, 1989), Social Exchange Theory (SET; Blau, 1956), and Psychological Ownership Theory (Pierce et al., 2003), we propose an overarching “vertical trust-affective channel-knowledge hiding” framework (see Figure 1) to explain how leader trust shapes employees' knowledge-hiding behavior in a knowledge-economy context and to highlight the boundary role of Knowledge Psychological Ownership (KPO). The theoretical logic and corresponding hypotheses are developed as follows.

### 3.1 Leader Trust and Knowledge Hiding: A Direct Inhibitory Path

Leader trust constitutes a high—quality social resource conveyed by managers, encompassing emotional support, developmental opportunities, and discretion. From a SET perspective, trust functions as a “social investment”: by signaling that subordinates are “worth investing in,” leaders trigger employees' reciprocity motivation. Concurrently, COR theory suggests that trust operates as a resource caravan passageway, alleviating employees' anxiety over the potential resource loss associated with sharing knowledge. In high-trust contexts, perceived failure risk is lower, making employees more willing to share tacit know-how and thereby reducing their propensity to hide knowledge. Accordingly, we hypothesize:

H1. Leader trust is negatively related to employee knowledge hiding.

### 3.2 Job satisfaction as an affective mediating pathway

Affective Events Theory (AET) posits that discrete workplace events trigger emotional reactions that subsequently shape employees' work attitudes and behaviors. Within this framework, leader trust represents a positive event that enhances employees' emotional arousal and sense of belonging, which is expected to translate into higher job satisfaction. Job satisfaction reflects employees' overall appraisal of the resources and opportunities provided by the organization; when satisfaction rises, employees are more likely to view the organization as a “resource-gain platform,” thereby attenuating the defensive mindset that fuels knowledge withholding. Prior research (e.g., Porter et al., 1974) further indicates that job satisfaction varies with contextual conditions and reliably predicts work performance and organizational citizenship behavior. Building on this logic, we propose the following hypotheses:

H2. Leader trust is positively associated with job satisfaction.

H3. Job satisfaction is negatively associated with knowledge hiding.

H4. Job satisfaction mediates the relationship between leader trust and knowledge hiding; that is, leader trust indirectly reduces knowledge hiding by enhancing job satisfaction.

### 3.3 Knowledge psychological ownership as a contextual moderator

Psychological ownership theory posits that when employees perceive knowledge as “mine,” they closely guard its flow and treat sharing as a potential resource loss (Peng, 2013). Under such circumstances, even explicit expressions of leader trust may not translate into higher affective investment or satisfaction, because employees' resource-defense motivation overrides reciprocity motives. Accordingly, high levels of Knowledge Psychological Ownership (KPO) are expected to dampen the positive affective response elicited by leader trust and, in turn, weaken the capacity of job satisfaction to curb knowledge hiding. Therefore, we advance the following hypotheses:

H5. Knowledge psychological ownership negatively moderates the positive relationship between leader trust and job satisfaction; specifically, the enhancing effect of leader trust on satisfaction becomes weaker when KPO is high.

H6. Knowledge psychological ownership negatively moderates the indirect effect of leader trust on knowledge hiding via job satisfaction; the higher the KPO, the weaker the mediating pathway.

## 4 Method

### 4.1 Research design and sample

A cross-sectional survey design was adopted. Data were collected from seventeen Chinese knowledge-intensive firms spanning four industries-IT services, pharmaceutical RandD, high-end equipment manufacturing and financial information consulting. Target firms were identified using industry-association directories and public annual reports. Within each firm, departments were first stratified into RandD, operations, and support functions, and employees were then randomly sampled across hierarchical positions (entry-level, middle management, and senior staff) to ensure representation across both functional and positional tiers. Participation was entirely voluntary and anonymity was guaranteed to reduce social-desirability bias.

To maximize content validity and contextual fit, a two-round Delphi process was conducted with a panel of 12 experts (six university researchers in organizational behavior and HRM, and six senior managers from knowledge-intensive firms). In Round 1, experts independently rated the clarity and relevance of each draft item on a 5-point scale; items with median ratings below 3.5 or an interquartile range above 1.0 were flagged for revision. In Round 2, experts reviewed the revised items and reached over 85% consensus on content adequacy. This process helped refine wording and ensure that scenario-based items (e.g., knowledge requests from colleagues vs. leaders) were both realistic and culturally appropriate.

A pilot test with forty two respondents yielded an exploratory factor analysis, leading to the elimination of items with low (< 0.50) or cross loadings. In the formal stage, 600 questionnaires were distributed and 556 returned. After removing cases with aberrant response time, failed attention checks, or logical inconsistencies, 518 valid responses remained (response rate = 86.7%).

The final sample comprised 45.4 % men and 54.6 % women, with an average age of 32.7 years (*SD* = 6.2). A total of 60.8 % held a bachelor's degree or higher, including 27.0 % with master's or doctoral degrees; mean organizational tenure was 3.8 years (*SD* = 2.9). Overall, the demographic profile mirrors China's knowledge-intensive workforce, supporting the sample's representativeness. Table 1 reports the respondents' profile.

**Table 1:** Demographic characteristics of the sample (*N* = 518).

**Variable**	**Category**	**Frequency**	**Percentage(%)**
**Gender**	Male	235	45.37
	Female	283	54.63
**Age**	≤ 25	109	21.04
	26–30	116	22.39
	31–40	105	20.27
	41–50	124	23.94
	≥51	64	12.36
**Education**	High school or below	46	8.88
	Junior college	157	30.31
	Bachelor's	175	33.78
	Master's	105	20.27
	Doctorate	35	6.76
**Organizational tenure**	< 1 year	109	21.04
	1–3 years	147	28.38
	4–5 years	108	20.85
	6–7 years	107	20.66
	>7 years	47	9.07

*Percentages may not sum to exactly 100 due to rounding*.

### 4.2 Measurement instruments

The final questionnaire comprised two sections. Section A captured six demographic variables (gender, age, education, organizational tenure, etc.). Section B contained 38 items measuring four focal constructs, each rated on a five-point Likert scale (1 = “strongly disagree,” 5 = “strongly agree”). Scale development employed a blind forward-back translation procedure. Two independent bilingual doctoral researchers with formal training in survey translation/psychometrics conducted the forward translation; a separate bilingual linguist with an M.A. in applied linguistics performed the back-translation. A bilingual professor of organizational behavior adjudicated discrepancies and confirmed semantic and contextual equivalence. Item sources and counts were as follows: leader trust (Gillespie, 2003; ten items), job satisfaction (Weiss et al., 1967; seventeen items), knowledge hiding (Connelly et al., 2012; ten items), and knowledge psychological ownership (Peng, 2013; three items). Reverse-scored items are flagged “^Ⓡ^”. Although the KPO scale included only three items, confirmatory factor analysis results indicated high reliability (Cronbach's α = 0.89; AVE = 0.73), which is adequate for our analytical approach (Table 2).

**Table 2:** Measurement items.

**Construct**	**Code**	**English item wording**	**Note**
Leader Trust (LT)	LT1	My immediate supervisor involves me in key decisions and allows me to exert influence.	
	LT2	My immediate supervisor pays appropriate attention to my work progress.	
	LT3	Even when unable to monitor me, my supervisor is comfortable assigning tasks to me.	
	LT4	My supervisor openly shares mistakes he or she has made at work.	
	LT5	My supervisor is willing to discuss contentious or unpopular views.	
	LT6^Ⓡ^	My supervisor worries that I might make things difficult for him/her at work.	Reverse
	LT7	When I raise questions, my supervisor provides detailed explanations.	
	LT8	If others question my motives, my supervisor chooses to believe me.	
	LT9	When I ask for assistance, my supervisor responds readily without weighing self-interest.	
	LT10	My supervisor frequently entrusts me with full responsibility for important projects.	
Job Satisfaction (JS)	JS1	I feel enthusiastic about my current job.	
	JS2	I maintain a high level of initiative at work.	
	JS3	This job fully utilizes my abilities.	
	JS4	I can play an important role within the team.	
	JS5	My work gives me a sense of achievement.	
	JS6	My job is fairly stable.	
	JS7	I often have opportunities to try different tasks.	
	JS8	I have opportunities to help other people.	
	JS9	I have authority to direct others in their work.	
	JS10	I enjoy autonomy and discretion in my position.	
	JS11	My work tasks do not conflict with my conscience.	
	JS12	Communication with my supervisor and colleagues is smooth.	
	JS13	I am satisfied with my supervisor's management style.	
	JS14	My income is generally commensurate with my effort.	
	JS15	I am satisfied with the policies the company formulates and implements.	
	JS16	There is a clear promotion path in my current work.	
	JS17	I am satisfied with the working environment and conditions.	
Knowledge Hiding (KH)	KH1^Ⓡ^	When a colleague requests information, I pretend not to know what he/she is talking about.	Reverse
	KH2^Ⓡ^	When a colleague seeks help, I claim to be unfamiliar with the topic.	Reverse
	KH3^Ⓡ^	Although I know the answer, I tell the colleague “I'm not sure.”	Reverse
	KH4^Ⓡ^	I divert the colleague to information unrelated to the issue.	Reverse
	KH5^Ⓡ^	I verbally agree to help but deliberately delay doing so.	Reverse
	KH6^Ⓡ^	I provide information that does not match the colleague's needs.	Reverse
	KH7^Ⓡ^	I refuse to share by stating that “the supervisor does not want this information disclosed.”	Reverse
	KH8^Ⓡ^	I state that the information is restricted to certain personnel only.	Reverse
	KH9^Ⓡ^	I explicitly say that I will not answer the colleague's question.	Reverse
	KH10^Ⓡ^	I feel I “should not reveal” relevant knowledge.	Reverse
Knowledge Psychological Ownership (KPO)	KPO1	I regard the knowledge I bring to work as my personal property.	
	KPO2	The knowledge I use at work belongs to me.	
	KPO3	The knowledge and experience I accumulate at work are owned by me personally.	

All items were rated on a 5-point Likert scale (1 = strongly disagree; 5 = strongly agree). Items marked “Ⓡ” are reverse-scored.

Pilot deletions (n = 42; principal axis factoring with Promax). Three pilot-only items were removed prior to CFA/SEM and therefore do not appear in Table 2:

JS-P1 (pilot-only): “I often have opportunities to try different tasks.” — primary loading < 0.50.

KH-P1 (pilot-only): “I divert the colleague to information unrelated to the issue.” — cross-loading > 0.40 on Job Satisfaction.

KH-P2 (pilot-only): “I provide information that does not match the colleague's needs.” — cross-loading > 0.40 on Job Satisfaction.

Table 2 reports the final retained items only.

## 5 Empirical results

### 5.1 Descriptive statistics and zero-order correlations

For the five hundred and eighteen valid cases, the mean scores (and standard deviations) were 3.62 (0.71) for leader trust, 3.55 (0.64) for job satisfaction, 3.21 (0.89) for knowledge psychological ownership, and 2.48 (0.83) for knowledge hiding. Pearson correlations (two-tailed) show that leader trust is negatively related to knowledge hiding (*r* = – 0.43, *p* < 0.01) and positively related to job satisfaction (*r* = 0.43, *p* < 0.01). Job satisfaction is negatively associated with knowledge hiding (*r* = – 0.35, *p* < 0.01), whereas knowledge psychological ownership is positively associated with knowledge hiding (*r* = 0.40, *p* < 0.01). These bivariate patterns provide preliminary support for the proposed paths (See Table 3).

**Table 3:** Means, standard deviations, and zero-order correlations (*N* = 518).

**Variable**	***M***	***SD***	**1**	**2**	**3**	**4**
1 Leader trust (LT)	3.62	0.71	—			
2 Job satisfaction (JS)	3.55	0.64	0.43^**^	—		
3 Knowledge hiding (KH)	2.48	0.83	−0.43^**^	−0.35^**^	—	
4 Knowledge psychological ownership (KPO)	3.21	0.89	−0.33^**^	−0.13^*^	0.40^**^	—

^*^p<*0.05*. ^**^p<*0.01 (two-tailed)*.

### 5.2 Measurement-model fit, reliability, and validity

The four-factor measurement model exhibits an excellent fit to the data (χ^2^/df = 1.98, CFI = 0.955, TLI = 0.948, RMSEA = 0.043, SRMR = 0.038) and outperforms three, two, and one-factor alternatives (see Table 5). Harman's single-factor test shows that the first factor accounts for only 28.4 % of the variance-well below the 40 % threshold-indicating that common-method bias is unlikely to be a serious concern. Internal consistency is satisfactory: Cronbach's α ranges from 0.86 to 0.91; composite reliability (CR) from 0.80 to 0.91; and average variance extracted (AVE) from 0.54 to 0.73-all exceeding conventional cut-offs, thereby supporting convergent validity (Table 4).

**Table 4:** Reliability and convergent-validity statistics.

**Construct**	**Items**	**Cronbach α**	**CR**	**AVE**
Leader trust	8	0.91	0.90	0.63
Job satisfaction	17	0.88	0.89	0.56
Knowledge hiding	10	0.86	0.86	0.54
Knowledge psychological ownership	3	0.89	0.89	0.73

**Table 5:** Fit indices for competing measurement models.

**Model**	**χ^2^/df**	**CFI**	**TLI**	**IFI**	**GFI**	**RMSEA**
Four-factor	1.98	0.955	0.948	0.957	0.959	0.043
Three-factor^2^(LT + JS)	2.03	0.879	0.829	0.849	0.924	0.098
Three-factor^2^(LT + KH)	2.24	0.846	0.804	0.826	0.805	0.117
Two-factor(LT + JS/KH + KPO)	3.94	0.764	0.618	0.592	0.614	0.123
Single-factor	4.25	0.640	0.575	0.579	0.562	0.137

Three-factor^2^ merges leader trust (LT) and job satisfaction (JS) because both capture employees' positive appraisals of leader–member exchange and supervisory treatment; this specification tests discriminant validity between two closely related relational constructs. Three-factor^2^ merges LT and knowledge hiding (KH); the two-factor model merges (LT + JS) and (KH + KPO); the single-factor model pools all four constructs.

### 5.3 Structural model and hypothesis testing

After controlling for gender, age, educational attainment, and organizational tenure, the structural equation model yielded the following results (Table 6). Leader trust exerted a significant negative effect on knowledge hiding (β = – 0.31, *p* < 0.001), supporting H1. Leader trust also had a significant positive effect on job satisfaction (β = 0.46, *p* < 0.001), while job satisfaction negatively predicted knowledge hiding (β = – 0.23, *p* < 0.001). Together, these two paths constitute a partial mediation; the bootstrap indirect effect was – 0.11 (95% CI [−0.17, −0.07]), accounting for 34 per cent of the total effect, thereby supporting H2–H4. In the moderated model, the interaction term (Leader Trust × Knowledge Psychological Ownership) exerted a significant negative effect on job satisfaction (β = −0.09, p = 0.004), indicating that knowledge psychological ownership (KPO) weakens the positive influence of leader trust on satisfaction. Johnson-Neyman analysis further revealed that when KPO is at or above M + 1.04 SD, the positive effect of leader trust on job satisfaction becomes non-significant, confirming H5.

**Table 6:** Direct and mediated effects.

**Path**	**β**	***SE***	***t/z***	***p***
LT → KH	−0.31	0.04	−7.51	< 0.001
LT → JS	0.46	0.04	11.44	< 0.001
JS → KH	−0.23	0.05	−4.39	< 0.001

The conditional indirect-effect analysis (5,000 bootstrap samples) shows that the mediating impact of job satisfaction diminishes as KPO increases: the indirect effect is −0.17 at low KPO (−1 SD) and declines to −0.06 at high KPO (+1 SD), with 95% confidence intervals excluding zero in all cases. These results corroborate H6 (Table 7).

**Table 7:** Conditional indirect effects at different levels of KPO (Bootstrap *N* = 5000).

**KPO Level**	**Indirect Effect**	**95% CI**
−1 SD	−0.17	[−0.24, −0.11]
Mean	−0.11	[−0.17, −0.07]
+1 SD	−0.06	[−0.10, −0.03]

### 5.4 Robustness checks

To assess the robustness of our findings, perceived organizational support and team competition climate were introduced as alternative control variables and the structural model was re-estimated. The coefficients on all key paths changed by less than 0.02, leaving the pattern of results intact. In addition, industry-specific subgroup analyses revealed that, within the pharmaceutical RandD subsample, the positive effect of leader trust on job satisfaction was even stronger (β = 0.54, *p* < 0.001), suggesting that vertical trust yields greater affective dividends in highly regulated, high-risk settings. Taken together, these supplementary tests consistently uphold the core “leader trust → job satisfaction → knowledge hiding” pathway and the attenuating role of knowledge psychological ownership.

## 6 Discussion and managerial implications

Drawing on five hundred and eighteen matched leader-employee surveys from the IT services, pharmaceutical RandD, high-end equipment manufacturing, and financial consulting sectors, this study empirically validates an affective-behavioral chain in which leader trust enhances job satisfaction, thereby suppressing knowledge hiding, and further demonstrates that knowledge psychological ownership (KPO) attenuates both the trust-satisfaction link and the overall indirect effect. By positioning vertical trust as an antecedent of knowledge hiding and integrating Conservation of Resources Theory with Social Exchange Theory, the findings shed light on the interplay between reciprocity motivation and resource-defense motivation, while recognizing that these contributions are incremental and should be interpreted in light of contextual boundaries.

### 6.1 Managerial implications

Cultivating a “high-trust-low-ownership” knowledge climate. Although vertical trust curbs knowledge hiding via greater satisfaction, this benefit weakens under high KPO conditions. Organizations can strengthen trust by delegated autonomy, mentoring, and timely feedback, while simultaneously reducing exclusivity through explicit authorship, patent-sharing bonuses, or team co-creation ceremonies. To ensure feasibility, firms may embed short “trust and satisfaction surveys” into existing HR routines rather than implementing resource-intensive systems. Simple dashboards that track changes in trust or ownership sentiments can guide timely yet low-cost managerial interventions.

Embedding satisfaction management into knowledge-management routines. Because job satisfaction bridges leader trust and knowledge behavior, managers should incorporate emotional check-ins into performance reviews or project debriefings. Feasible initiatives include peer-support groups, team reflection meetings, or recognition programs. Linking satisfaction with knowledge contribution (e.g., cross-departmental showcases or redeemable training credits) can create a cycle of “satisfaction → sharing → further satisfaction” without placing excessive financial burdens on firms.

Deploying targeted interventions for high-KPO employees. Employees with strong ownership feelings may resist sharing despite being trusted. Instead of costly analytics, firms can rely on existing performance data or project records to identify such employees. Recognition strategies—such as mentoring roles, expert titles, or newsletter features-help affirm identity while positioning them as contributors to collective goals. Cross-functional projects and collaborative platforms can highlight the collective benefits of sharing and gradually reduce possessive impulses.

### 6.2 Theoretical implications

This study contributes to the literature in three moderated ways. First, by examining direct supervisor trust, we highlight the underexplored role of vertical trust alongside the more frequently studied peer trust. Second, by integrating Conservation of Resources Theory (COR) and Social Exchange Theory (SET), we illustrate how leader-provided socio-emotional resources activate reciprocity, while high psychological ownership triggers resource-defense, revealing a dynamic but context-dependent balance between the two logics. Third, rather than treating KPO only as a direct antecedent, we clarify its boundary role: under high KPO, the indirect effect of leader trust on knowledge hiding through job satisfaction declines from – 0.17 to – 0.06. These findings extend but do not redefine existing theories, and should be interpreted as a step toward a more nuanced understanding.

### 6.3 Practical implications

The study yields three actionable insights. First, the “leader trust → job satisfaction → knowledge hiding” pathway suggests that enabling employees to feel trusted is a practical entry point to reduce defensive knowledge behaviors. Second, managers should balance recognition of individual knowledge contributions with mechanisms that prevent exclusivity-such as formal authorship or shared rewards-to maintain fluid knowledge flow. Third, feasible monitoring tools (e.g., simple HR dashboards or regular team surveys) can alert managers to declining trust or rising ownership sentiments. These tools should remain proportionate to organizational resources to ensure sustainability.

### 6.4 Limitations and directions for future research

Although this study employed a rigorous design and a sizable sample, several limitations remain. The reliance on self-reported, cross-sectional data makes it difficult to completely rule out concerns of common method bias and reverse causality, even though statistical checks suggested these issues were not severe. In addition, the Chinese cultural context in which the data were collected may constrain the extent to which the findings can be generalized to other institutional environments, particularly Western settings where leader-employee dynamics and knowledge norms may differ. Another limitation lies in the voluntary nature of participation, which raises the possibility of selection bias, as employees more comfortable with sharing may have been more willing to respond.

Beyond these general concerns, several more specific issues warrant attention. One relates to the leader-employee dyad. This study focused on direct supervisor-employee interactions, but hierarchical distance-such as whether an employee interacts with an immediate supervisor or a higher-level manager-may influence the development of trust and the manifestation of knowledge behaviors. Another concerns the type of knowledge itself. We did not differentiate between product-related (tangible) and process-related (intangible) knowledge, nor between explicit and tacit knowledge. Since psychological ownership is often stronger for tacit forms, its role may extend beyond that of a moderator to exert direct effects on knowledge hiding. Finally, although we controlled for organizational tenure, we did not capture the duration of the specific leader-employee relationship. Relational length and congruence in attributes such as age, education, and professional background could further condition how trust translates into satisfaction and subsequent knowledge-sharing or hiding behaviors.

Looking ahead, future research should address these limitations by employing longitudinal or experience-sampling designs that can capture the dynamic interplay of trust, satisfaction, and knowledge hiding over time, thereby providing stronger evidence of causality. Cross-cultural replications and multi-industry studies would be valuable for testing the robustness of the mechanisms identified here and for extending the findings beyond the Chinese context. To refine our understanding of dyadic factors, subsequent studies should explicitly model the influence of hierarchical distance on trust-satisfaction-hiding pathways. Likewise, distinguishing between explicit and tacit knowledge, and between product and process knowledge, would allow scholars to test whether psychological ownership operates differently across knowledge domains and whether it simultaneously exerts direct and moderating effects. Finally, by incorporating indicators of the length and quality of leader-employee relationships, as well as demographic and professional congruence, future research could provide more nuanced insights into how relational histories shape employees' willingness to share or conceal knowledge.

## Data Availability

The raw data supporting the conclusions of this article will be made available by the authors, without undue reservation.
